# Genetic locus responsible for diabetic phenotype in the insulin hyposecretion (*ihs*) mouse

**DOI:** 10.1371/journal.pone.0234132

**Published:** 2020-06-05

**Authors:** Kenta Nakano, Rieko Yanobu-Takanashi, Yukiko Shimizu, Yuki Takahashi, Koki Hiura, Masaki Watanabe, Hayato Sasaki, Tadashi Okamura, Nobuya Sasaki

**Affiliations:** 1 Department of Laboratory Animal Medicine, Research Institute, National Center for Global Health and Medicine (NCGM), Shinjuku, Tokyo, Japan; 2 Laboratory of Laboratory Animal Science and Medicine, School of Veterinary Medicine, Kitasato University, Towada, Aomori, Japan; 3 Department of Pediatrics and Adolescent Medicine, Juntendo University Graduate School of Medicine, Bunkyo, Tokyo, Japan; 4 Section of Animal Models, Department of Infectious Diseases, Research Institute, National Center for Global Health and Medicine, Shinjuku, Tokyo, Japan; Centre de Recherche des Cordeliers, FRANCE

## Abstract

Diabetic animal models have made significant contributions to understanding the etiology of diabetes and to the development of new medications. Our research group recently developed a novel diabetic mouse strain, the insulin hyposecretion (*ihs*)mouse. The strain involves neither obesity nor insulitis but exhibits notable pancreatic β-cell dysfunction, distinguishing it from other well-characterized animal models. In *ihs* mice, severe impairment of insulin secretion from pancreas has been elicited by glucose or potassium chloride stimulation. To clarify the genetic basis of impaired insulin secretion, beginning with identifying the causative gene, genetic linkage analysis was performed using [(C57BL/6 × *ihs*) F_1_ × *ihs*] backcross progeny. Genetic linkage analysis and quantitative trait loci analysis for blood glucose after oral glucose loading indicated that a recessively acting locus responsible for impaired glucose tolerance was mapped to a 14.9-Mb region of chromosome 18 between *D18Mit233* and *D18Mit235* (the *ihs* locus). To confirm the gene responsible for the *ihs* locus, a congenic strain harboring the *ihs* locus on the C57BL/6 genetic background was developed. Phenotypic analysis of B6.*ihs*-(*D18Mit233*-*D18Mit235*) mice showed significant glucose tolerance impairment and markedly lower plasma insulin levels during an oral glucose tolerance test. Whole-genome sequencing and Sanger sequencing analyses on the *ihs* genome detected two *ihs*-specific variants changing amino acids within the *ihs* locus; both variants in *Slc25a46* and *Tcerg1* were predicted to disrupt the protein function. Based on information regarding gene functions involving diabetes mellitus and insulin secretion, reverse-transcription quantitative polymerase chain reaction analysis revealed that the relative abundance of *Reep2* and *Sil1* transcripts from *ihs* islets was significantly decreased whereas that of *Syt*4 transcripts were significantly increased compared with those of control C57BL/6 mice. Thus, *Slc25a46*, *Tcerg1*, *Syt4*, *Reep2* and *Sil1* are potential candidate genes for the *ihs* locus. This will be the focus of future studies in both mice and humans.

## Introduction

Type 2 diabetes (T2D) is a metabolic disorder characterized by abnormal glucose homeostasis due to a defect in the secretion and/or action of insulin. T2D affects more than 200 million individuals worldwide, constituting a major public health problem globally, and its prevalence is increasing in many countries [1]. T2D shows different pathologies depending on race. In general, T2D in East Asian countries is characterized by lower levels of insulin secretion from pancreatic islets and more frequently a non-obese type, differing from its pattern among Caucasians [2–5]. The etiology of T2D is complicated, involving multiple genetic and environmental factors [6]. Therefore, there has been increased interest in animal models of T2D which genetic and environmental factors that could influence the development of the disease and related complications can be precisely controlled in vivo. Animal models have made significant contributions to the study of diabetes mellitus, providing new information on its management and treatment in humans [7, 8].

Recently, our research group established a novel, non-obese mouse strain with spontaneous diabetes, called the insulin hyposecretion (*ihs*) mouse [9]. During the oral glucose tolerance test (OGTT), *ihs* mice exhibit markedly impaired glucose tolerance caused by lower concentrations of plasma insulin, whereas their insulin sensitivity and insulin synthetic ability are found to be normal. The *ihs* mice show a severe defect in insulin secretion from pancreatic islets even when the islets are stimulated by potassium chloride (KCl) and sulfonylurea, which can directly cause β-cell plasma membrane depolarization followed by an influx of Ca^2+^ and insulin granule exocytosis [10, 11]. These results indicate that the insulin secretory disorder in the *ihs* mouse is caused by a defect in the Ca^2+^ signaling pathway in β-cells [9]. Thus, these characteristics distinguish *ihs* mice from other well-characterized T2D animal models. Therefore, the *ihs* mouse offers the promise of identifying of a novel gene involved in insulin secretion through regulation of the Ca^2+^ signaling pathway.

In this study, we performed genetic linkage analysis and quantitative trait loci (QTL) analysis of the glucose tolerance phenotype as a first step toward identifying the gene responsible for the impaired insulin secretion of *ihs* mice. Furthermore, we developed and analyzed a novel congenic mouse strain harboring the *ihs* locus on a C57BL/6 genetic background to confirm the *ihs* locus identified by genetic linkage analysis.

## Materials and methods

### Ethical statement

All animal experiments were approved by the President of NCGM and Kitasato University, following consideration by the Institutional Animal Care and Use Committee of NCGM (approval ID: no. 17056) and Kitasato University (approval ID: no. 17–099), and were carried out in accordance with institutional procedures, national guidelines, and the relevant national laws on the protection of animals.

### Animals

The *ihs* mice were established at the School of Veterinary Medicine at Kitasato University, maintained at the Research Institute at the NCGM, and deposited at the Biological Resource Center of National Institute of Technology and Evaluation (Chiba, Japan) (deposit number, NITE P-02377). C57BL/6NCr (B6) mice were purchased from Japan SLC (Hamamatsu, Japan). All mice used in this study were housed in an air-conditioned animal room at 23 ± 2°C with relative humidity of 40%−60% under specific-pathogen-free (SPF) conditions, with a 12-h light/dark cycle (8:00−20:00/20:00−8:00). All mice were fed a standard rodent CE-2 diet (CLEA Japan, Tokyo, Japan) and had *ad libitum* access to water.

### Oral glucose tolerance test (OGTT)

Only male mice were used in this study, because the glucose intolerance is more prominent in males than in females [9]. The mice were fasted for 16 h, and tail blood glucose was measured at 0, 30, 60, 90, and 120 min after oral administration of glucose (2 g/kg body weight; Otsuka Pharmaceutical, Tokyo, Japan) by gavage. The blood glucose level was determined using Glutest Ace (Sanwa Kagaku Kenkyusho Co, Nagoya, Japan). For measurement of plasma insulin levels, plasma samples were collected from the retro-orbital venous plexus at 0, 15, and 30 min during OGTT. Plasma insulin levels were measured using a mouse insulin kit (Morinaga Institute of Biological Science, Inc., Kanagawa, Japan). Each area the under curve (AUC) of the blood glucose and insulin in the OGTT was calculated by the trapezoidal rule from glucose measurements at 0, 30, 60, 90, and 120 min and from plasma insulin measurements at 0, 15 and 30 min [9].

### Genetic mapping

To enable genetic mapping for the *ihs* locus, F_1_ progeny were generated by crossing male *ihs* mice with female B6 mice. Both male and female F_1_ progeny were then backcrossed to *ihs* mice to produce N_2_ progeny. Genomic DNA samples from male N_2_ progeny were extracted from a tail clip by standard phenol/chloroform extraction [9] and were genotyped using polymorphic 59 simple sequence length polymorphism (SSLP) markers (S1 Table) as described previously [12]. The map positions of the SSLP and single nucleotide polymorphism (SNP) markers were established based on a mouse genome, Build GRCm38. In total, 96 male N_2_ progeny were genotyped for the *ihs* locus according to their blood glucose levels measured at 60 min during OGTT. Mice with blood glucose levels less than 250 mg/dl at 60 min after the oral administration of glucose were taken to be normal glucose tolerance (NGT) type, and mice with blood glucose levels of 250 mg/dl or more at 60 min after the oral administration of glucose were considered the impaired glucose tolerance (IGT) type. The association between the *ihs* locus and each SSLP marker was individually evaluated via a chi-squared test using JMP7 statistics software (SAS Institute Japan, Tokyo, Japan) [12]. QTL analysis for blood glucose was performed using Map Manager QTXb20, a software program that uses a maximum likelihood algorithm with interval mapping and simultaneous search and that permits improved localization of loci and exclusion mapping [13]. Recombination frequencies (as percentages) were converted into genetic distance (centimorgans, cM) using the Kosambi map function. For each chromosome, a likelihood ratio statistic (LRS) score was calculated using 1000 random permutations of the trait values relative to the genotypes of the marker loci. Suggestive and significant threshold levels were calculated using 1000 random permutation tests, based on already-established guidelines [14]. Blood glucose levels at 60 min after the oral glucose administration were used as the quantitative trait. QTL data also were confirmed using QTL Cartographer 2.5 [15].

### Development of congenic strain

To introgress the *ihs* locus derived from *ihs* mice into B6 mice, the F_1_ (*ihs *× B6) progeny were backcrossed to B6 mice for 12 generations. The homozygous B6.*ihs*-(*D18Mit233*-*D18Mit235*) (hereafter, B6-*ihs*) were subsequently produced and maintained by sib-mating. The primer sets used to create B6-*ihs* are listed in S2 Table.

### Whole-genome sequencing and candidate gene search

DNA was isolated from tail clips of female *ihs* mice using NucleoSpin Gel and PCR Clean-up kit (Takara Bio Inc., Shiga, Japan). A DNA library for the whole-genome sequence (WGS) was then prepared using NEBNext UltraTM DNA Library Prep kit (Illumina, San Diego, CA, USA), following the manufacturer's instructions. Prepared libraries were sequenced in paired-end 150-nucleotide reads using an Illumina NovaSeq 6000 (Illumina). Sequencing and library construction were performed by GENEWIZ (South Plainfield, NJ, USA). Data were aligned with the Burrows-Wheeler Alignment tool [16]; the SNV/InDel was selected using the Genome Analysis Toolkit pipeline (Broad Institute, Cambridge, MA, USA) [17], and was annotated by ANNOVAR [18]. The WGS data were registered with the DNA Data Bank of Japan (accession no. DRA010083). The variants were confirmed as not already present in the mouse genetic variation database (the Mouse Genome Project, a reference database of genetic variations in laboratory mice). An amino acid substitution that affects protein function was predicted by the online software tools SIFT [19] and PROVEAN [20].

For Sanger sequencing, genomic DNAs from *ihs*, B6 and ICGN mice were amplified with the gene-specific primer sets listed in S3 Table. The PCR products were purified using ExoSAP-IT Express (Thermo Fisher Scientific, Waltham, MA, USA) and used as templates in the sequence reaction. Sequencing was performed using BigDye terminator v3.1 cycle sequencing kit (Thermo Fisher Scientific) and ABI 3130xl Genetic Analyzer (Thermo Fisher Scientific).

### Reverse-transcription quantitative polymerase chain reaction (RT-qPCR)

Pancreatic islets were isolated from the pancreas by collagenase digestion as previously described [21]. Briefly, male mice at age 10 weeks were anesthetized with Sevoflurane (Mylan, Inc., Southpoint, PA, USA), followed by injection of collagenase into the common bile duct. The pancreas was removed by dissection and was incubated at 37°C for 12 min. The islets were then collected under a stereoscopic microscope. Total RNAs from islets were extracted with the Isogen RNA extraction kit (Nippon Gene, Tokyo, Japan), and cDNA was prepared with ReverTra Ace (Toyobo, Osaka, Japan). For quantification, gene-specific primers (S4 Table) were used with Thunderbird SYBR qPCR Mix (Toyobo, Osaka, Japan) in a 7900HT Fast Real-Time PCR System (Applied Biosystems, Foster City, CA, USA). The expression level of each target gene was normalized to the level of *Actb* mRNA. Each experiment was performed in three biological replicates.

### Statistics

The data are expressed as means ± standard error. Student’s *t*-test was used for comparisons of two independent groups. The results for blood glucose levels and plasma insulin levels during OGTT were analyzed using repeated-measures analysis of variance (ANOVA). A *P* value <0.05 was considered statistically significant.

## Results

### The mode of inheritance of insulin hyposecretion in *ihs* mice

**Fig 1:**
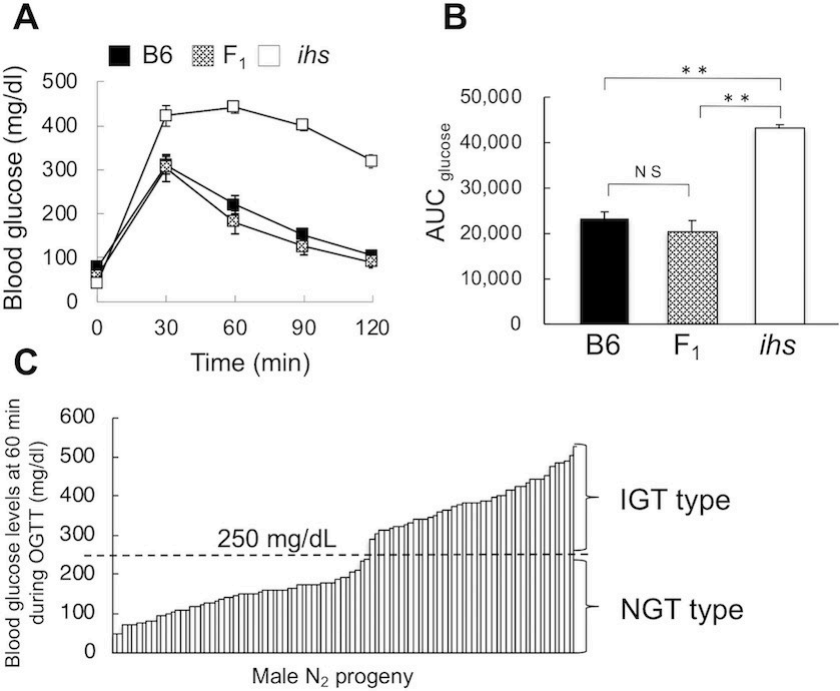
The oral glucose tolerance test in (B6 × *ihs*) F_1_ and [(B6 × *ihs*) F_1_× *ihs*] N_2_ progeny. a. Plot of the individual blood glucose (mg/dl) of *ihs* (n = 6), B6 (n = 6), and F_1_ mice (n = 5) at 10–15 weeks of age. The blood glucose levels were measured at 0, 30, 60, 90, and 120 min after the oral glucose administration. b. AUC of the glycemic responses for 0–120 min. AUC of glucose was calculated using the trapezoidal rule. ** *P* < 0.01, NS: not significant. c. Distribution of blood glucose levels in 96 N_2_ progeny at 60 min after the oral glucose administration. The vertical dotted line indicates the border of the phenotype as defined in the Material and Methods section. IGT: impaired glucose tolerance, NGT: normal glucose tolerance.

In a previous study, our research group demonstrated that *ihs* mice showed marked impaired glucose tolerance due to impaired insulin secretion [9]. Therefore, the blood glucose levels after glucose loading were adopted as an indicator of impaired insulin secretion. To determine the mode of inheritance of insulin hyposecretion in *ihs* mice, F_1_ and N_2_ progeny were generated, and OGTTs were performed in these mice. As demonstrated in a previous study, *ihs* mice showed a remarkable impairment of glucose tolerance. However, the glucose tolerance of F_1_ mice was comparable to that of B6 mice (Fig 1A). The glucose AUC also showed no statistically significant difference between F_1_ and B6 mice (F_1_: 20,499.0 ± 2,331.5, vs. B6: 23,197.5 ± 1,358.5, *P* = 0.35, Fig 1B). Although the blood glucose levels at 60 min after glucose administration in the N_2_ progeny showed consecutive distribution (Fig 1C), there seemed to be a distinct threshold to be able to be divided into two groups. Based on the result of OGTTs in B6, *ihs* and F_1_ mice (Fig 1A), we divided them into two groups (NGT and IGT type) at 250 mg/dl (Fig 1C). In contrast, it was difficult to divide those of 120 min after glucose administration into groups due to a continuous trait (S1 Fig). Those N_2_ progeny with blood glucose levels less than 250 mg/dl at 60 min after the oral administration of glucose were labeled the NGT type, and those N_2_ progeny with blood glucose levels of 250 mg/dl or more at 60 min after the oral administration of glucose were called the IGT type. Of the 96 male offspring, 53 (55.2%) and 43 (44.8%) individuals at 10–15 weeks of age were classified as NGT and IGT type, respectively, indicating that hyposecretion of insulin in *ihs* mice is inherited in a recessive fashion (Fig 1C).

### Genetic linkage analysis of *ihs* locus

**Fig 2:**
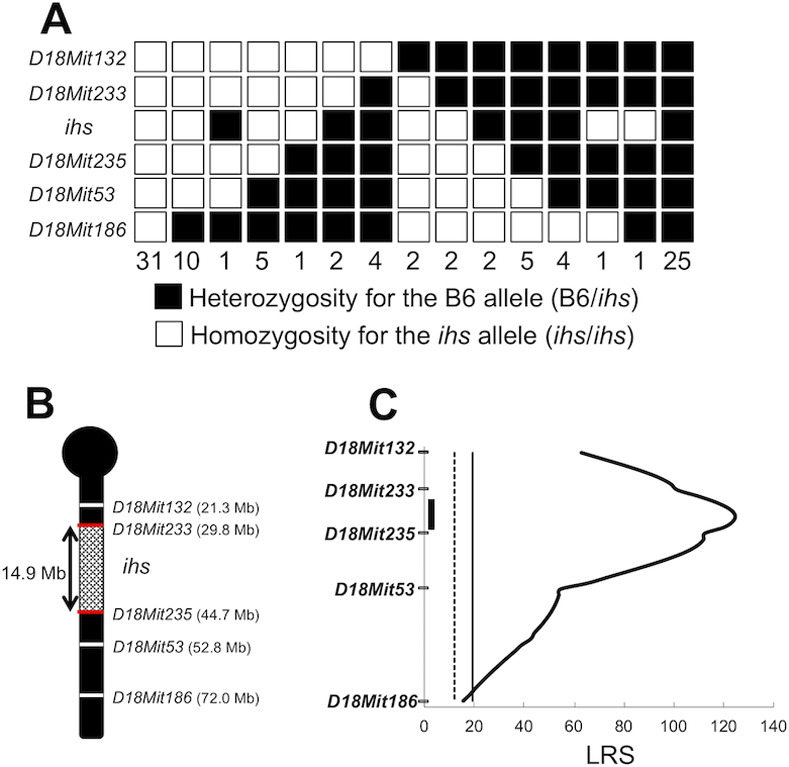
Genetic mapping of the *ihs* locus. a. The haplotype analysis of *ihs* locus on chromosome 18 using 96 N_2_ progeny. A black box indicates the heterozygosity for the B6 allele (B6/*ihs*). A white box indicates the homozygosity for the *ihs* allele (*ihs*/*ihs*). The number of N_2_ progeny with each genotype is listed at the bottom of each column. b. Schematic diagram of the *ihs* locus. The region of the *ihs* locus is shown by braiding. c. LRS score plot for QTL of blood glucose at 60 min after oral glucose administration in 96 N_2_ progeny. One highly significant peak (LRS score 124.5) is located on chromosome 18. The vertical solid line (LRS score 19.4) and dotted line (LRS score 12.1) indicate highly significant and significant threshold levels for the LRS score, respectively. The vertical black bar indicates the 95% confidence interval.

To identify the locus responsible for impaired insulin secretion in *ihs* mice (*ihs* locus), DNA samples were genotyped from 96 male N_2_ progeny using 59 SSLP markers, in order to cover the whole mouse genome (S1 Table). The association could then be examined between the genotype of each SSLP marker and the phenotype, which had already been defined as either the B6 or the *ihs* type, in terms of glucose tolerance for each individual. Using a chi-squared test, a significant association was detected between the *ihs* locus and SSLP markers on chromosomes 4, 6, 18, 19, and X, with an especially highly significant association at *D18Mit233* (*P* = 1.78E-18) (Table 1). Since it was possible that the *ihs* locus was located on chromosome 18, haplotype analysis was performed using additional three SSLP markers on chromosome 18. This haplotype analysis revealed that the *ihs* locus was located around an interval of 14.9-Megabase (Mb) between *D18Mit233* and *D18 Mit235* (Fig 2A and 2B).

**Table 1:** SSLP markers that show significant association between genotype and glucose tolerance.

Chr	Locus name	Position	Normal glucose tolerance^b^		Impaired glucose tolerance^c^		*χ*^*2*^	*P* value
		(Mb)^a^	*ihs/ihs*	B6/*ihs*	*ihs/ihs*	B6/*ihs*		
4	*D4Mit13*	142.5	18	35	25	18	5.61	0.02
6	*D6Mit69*	83.7	32	21	24	19	0.65	0.05
18	*D18Mit233*	29.8	3	50	41	2	76.9	1.78E-18
18	*D18Mit53*	52.8	8	45	36	7	45.0	1.94E-11
19	*D19Mit1*	54.9	17	36	24	19	5.47	0.02
X	*DXMit186*	165.4	18	35	25	18	5.61	0.02

Chi-squared test results with *P* values less than 0.05 are shown. A genome-wide scan was performed in 96 N_2_ progeny using 59 SSLP markers as shown in S1 Table.

a. GRCm38.

b. N_2_ progeny showing blood glucose levels less than 250 mg/dl at 60 min after the oral administration of glucose (2 g/kg body weight) by gavage.

c. N_2_ progeny showing blood glucose levels of 250 mg/dl or more at 60 min after the oral administration of glucose (2 g/kg body weight) by gavage.

Since blood glucose is considered a measurable phenotype that depends on the cumulative actions of many genes and the environment, QTL analysis was performed for blood glucose levels at 60 min after the oral glucose administration. One significant QTL peak was identified on chromosome 18 between *D18Mit233* and *D18Mit235* with a likelihood ratio statistic (LRS) score of 124.5 (Table 2 and Fig 2C), and there were two suggestive QTL peaks on chromosome 4 at *D4Mit13* and chromosome 11 at *D11Mit38* with LRS scores of 7.2 and 8.3, respectively (Table 2). There were no QTL peaks beyond the significant threshold level (LRS = 12.1) on any other chromosome except for chromosome 18 (Tables 2 and S5). In addition, QTL analysis for blood glucose levels at 120 min after glucose administration also showed a highly significant QTL peak between *D18Mit132* and *D18Mit53* on chromosome 18 (S6 Table) and a significant QTL peak at *D11Mit38* on chromosome 11.

**Table 2:** SSLP markers that show an LRS peak beyond the suggestive threshold level.

Chr	Locus name	Position	blood glucose (mg/dl) ^b^		*P* value ^c^	LRS ^d^
		(Mb) ^a^	*ihs*/*ihs*	B6/*ihs*		
4	*D4Mit13*	142.5	292.6±21.3	219.7±17.1	8.14E-03	7.2
11	*D11Mit38*	56.3	292.9±20.8	215.1±17.0	4.45E-03	8.3
18	*D18Mit132*	21.3	354.3±14.1	176.4±15.1	5.93E-13	63.0
18	*D18Mit233*	29.8	370.9±13.0	152.1±10.5	2.70E-23	101.5
18	*D18Mit235*	44.7	376.8±12.7	151.4±9.5	1.22E-25	112.4
18	*D18Mit53*	52.8	348.9±16.8	170.7±13.2	3.20E-13	54.5
18	*D18Mit186*	72.0	306.6±20.4	199.0±15.3	9.55E-05	15.6

The SSLP markers with likelihood ratio statistics (LRS) more than 6.2 are shown.

a. GRCm38.

b. Blood glucose levels at 60 min during OGTT.

c. Statistical difference between genotypes (*ihs*/*ihs* vs. B6/*ihs*) calculated by Student's *t*-test.

d. Likelihood ratio statistics (LRS) were calculated using Map Manager QTXb20. The suggestive, significant, and highly significant threshold levels of LRS score are 6.2, 12.1, and 19.4, respectively.

### Glucose tolerance and insulin secretion of B6.*ihs*-(*D18Mit233-D18Mit235*) during the oral glucose tolerance test

**Fig 3:**
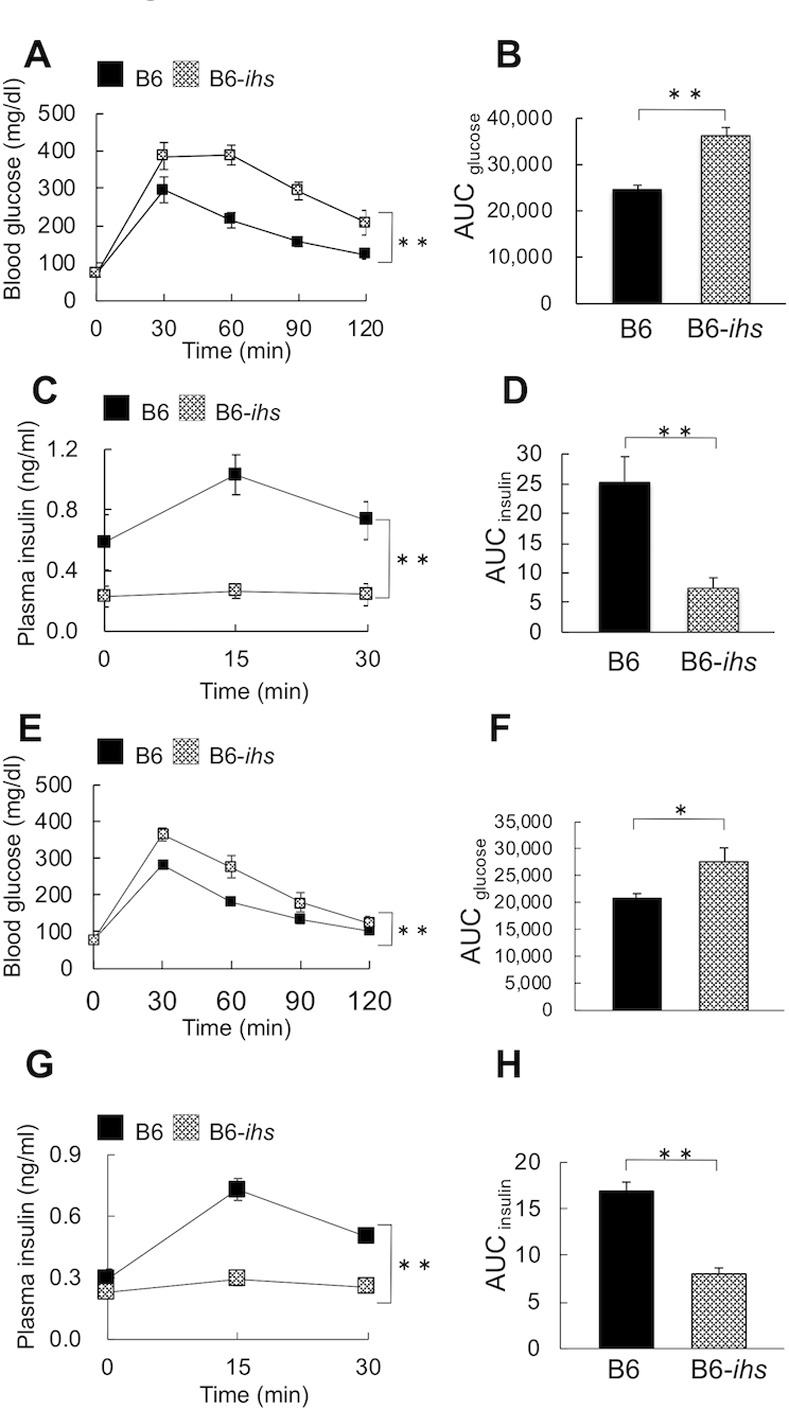
The glucose tolerance and insulin secretion of B6-*ihs* mice during OGTT. a, e. Plot of the blood glucose (mg/dl) of B6-*ihs* (male; n = 6, female; n = 9) and B6 (male; n = 6, female; n = 9) mice at 9–12 weeks of age. The blood glucose levels were measured at 0, 30, 60, 90, and 120 min after the oral glucose administration. b, f. The area under the curve (AUC) of the 0–120 min glycemic responses. c, g. The plasma insulin levels of B6-*ihs* (male; n = 5, female; n = 8) and B6 (male; n = 5, female; n = 7) mice at 10–17 weeks of age during OGTT. The plasma insulin levels were measured at 0, 15, and 30 min after glucose loading. d, h. AUC for insulin during 0–30 min. AUC was calculated using the trapezoidal rule. * *P* < 0.05, ** *P* < 0.01.

To confirm that the gene responsible for the *ihs* locus (the *ihs* gene) was present in the region around between *D18Mit233* and *D18Mit235*, a congenic mouse (B6-*ihs*), was produced by introgression of the region between *D18Mit233* and *D18Mit235* from the *ihs* mouse on the B6 genetic background. Upon OGTT, the B6-*ihs* mice showed marked impaired glucose tolerance compared to B6 mice (Fig 3A). The glucose AUC values after glucose loading in the B6-*ihs* mice also were higher than those of the B6 mice (B6-*ihs*: 36,366 ± 1,737.7 vs. B6: 24,594 ± 980.3, *P* < 0.01, Fig 3B). Furthermore, markedly lower plasma insulin levels during OGTT was also observed in B6-*ihs* mice (Fig 3C). The AUC for plasma insulin levels of *ihs* mice was significantly lower than those of B6 mice (B6-*ihs*: 7.49 ± 1.68 vs. B6: 25.37 ± 4.17, *P* < 0.01, Fig 3D). Female B6-*ihs* mice also showed impaired glucose tolerance and lower plasma insulin concentration than B6 mice during OGTT (Fig 3E, 3F, 3G and 3H). These results demonstrate that the *ihs* gene was definitely present in the region from *D18Mit233* to *D18Mit235*, a region that contained 199 genes.

### Whole-genome sequencing and candidate gene search

To identify the *ihs* mutation, WGS analysis was applied to the *ihs* genome to detect the differences between the *ihs* genome and the reference sequence (GRCm38) of the B6J strain. Non-synonymous single nucleotide variants (nsSNVs) and/or insertion deletion (InDels) were sought that fulfilled the following four criteria: 1) nsSNVs and/or InDels in the coding regions of genes that were located within the *ihs* locus, 2) those that were homozygous genotype, because *ihs* mice had an autosomal recessive disorder, and 3) those that were *ihs*-specific variants, which were confirmed as not being in the mouse genetic variation databases, because hypoinsulin secretion from islets is a striking characteristic of the *ihs* strain, and 4) those that were confirmed by Sanger sequencing. The variants that fulfilled these criteria were evaluated as candidates for *ihs* mutation(s). WGS analysis of *ihs* genome revealed that 28,970 nsSVNs existed within the *ihs* locus (S7 Table), and the two *ihs*-specific nsSVNs in *Slc25a46* and *Tcerg1* genes satisfied the four criteria just described (Table 3). These nsSNVs were not present in either the dbSNP database or the 36 common inbred strains genome database in the Sanger Center (https://www.sanger.ac.uk/science/data/mouse-genomes-project). In addition, there were 10,442 InDels within the *ihs* locus (S8 Table); however, no InDel satisfied the criteria. Although two nsSNVs (c.A537C and c.T539C) and two InDels (c.527_529delACT and c.541_542insTAC) in *Pcdhgb7* were identified by WGS analysis, these variants changing amino acids could not be confirmed by Sanger sequencing, indicating that the nsSNVs and InDels in *Pcdhgb7* were detected by a WGS sequence error and/or errors due to the parameters for the SNP and InDel labeling used for data analysis.

**Table 3:** nsSNVs and their impact for amino acid changes in the *ihs* locus.

Gene Symbol		*Slc25a46*	*Tcerg1*
nsSNVs/ InDels		c.A683G	c.C1586T
Alteration of amino acid		p.N228S	p.T529I
Nucleic acid sequence of each strain ^a^	*ihs*	G	T
	ICGN	A	T
	B6	A	C
Amino acid conservation	Human	N	A
	Chimpanzee	N	A
	Dog	N	T
	Cow	N	T
	Mouse	N	T
	Rat	N	T
Effect on protein	SIFT	Tolerated	Tolerated
	PROVEAN	Deleterious	Deleterious
Gene ID: MGI		1914703	1926421
Gene ID: NCBI		67453	56070

a. Sequence was confirmed by Sanger sequencing.

Since the *ihs* mice were derived from the ICGN mice, we compared the variants changing amino acids found in the *ihs* mice with those in ICGN mice. The c.A683G in *Slc25a46* was detected only in the *ihs* genome, whereas the c.C1586T in *Tcerg1* was confirmed in both the *ihs* and ICGN genome (Table 3).

In terms of *in silico* prediction on whether an amino acid substitution has an impact on the biological function of a protein, PROVEAN [20] predicted a “deleterious” effect of p.N228S in *Slc25a46* and of p.T529I in *Tcerg1*, and these effects are believed to disrupt the function of the protein, whereas SIFT [19] suggested that both p.N228S in *Slc25a46* and p.T529I in *Tcerg1* have little effect on these protein function (Table 3).

**Fig 4:**
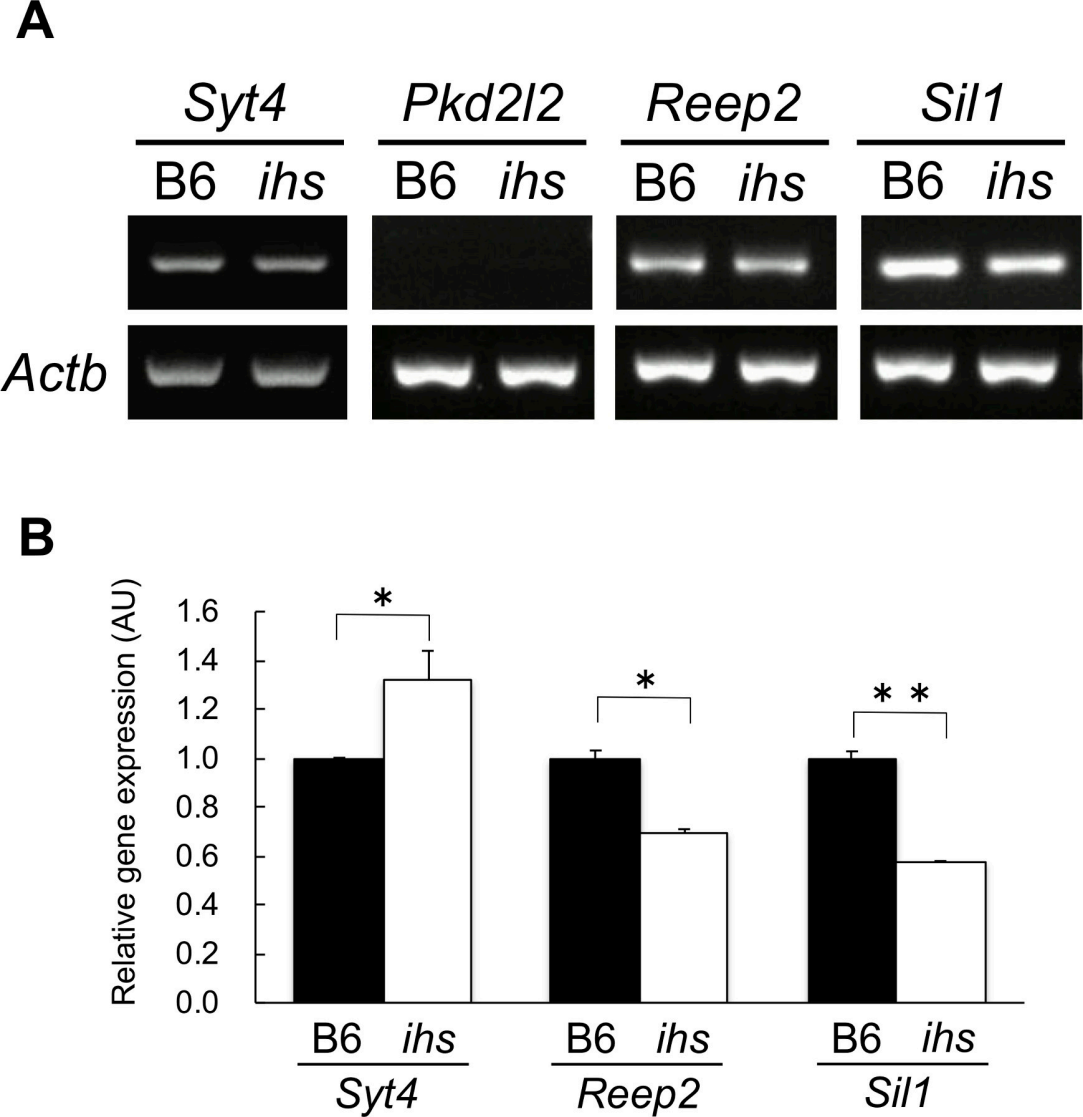
Comparison of the expression levels of candidate genes in pancreatic islets from B6 and *ihs* mice. a. RT-PCR analysis for *Syt4*, *Pkd2l2*, *Reep2*, and *Sil1*. b. RT-qPCR analysis of *Syt4*, *Reep2*, and *Sil* gene in B6 (closed bars; n = 3) and *ihs* islets (open bars; n = 3) at 10 weeks of age. The values were arbitrary units after normalization against *Actb*. Each experiment was carried out in three biological replicates. *P < 0.05, **P < 0.01.

In addition, given the information regarding the candidate genes for the *ihs* locus based on PubMed and MGI database, synaptotagmin 4 (*Syt4*), polycystic kidney disease 2 like 2 (*Pkd2l2*), receptor accessory protein 2 (*Reep2*), and endoplasmic reticulum chaperone SIL1 homolog (*Sil1*), which have been found involved in diabetes mellitus and/or insulin secretion, were the candidate genes for the *ihs* locus. However, WGS analysis of the *ihs* genome indicated that there were no nsSNVs and InDels in the protein coding regions of *Syt4*, *Pkd2l2*, *Reep2*, and *Sil1*genes (S7 and S8 Tables). Therefore, the mRNA expression of candidate genes in the pancreatic islets from B6 and *ihs* mice was next examined via RT-qPCR analysis. The relative abundance of *Reep2* and *Sil1* transcripts from the *ihs* islets were 1.4-fold and 1.7-fold lower than those from B6 islets, respectively, whereas the abundance of *Syt4* transcripts was 1.3-fold higher than for those from B6 (Fig 4B). The *Pkd2l2* transcripts could not be detected in either *ihs* or B6 islets (Fig 4A). In summary, *Slc25a46*, *Tcerg1*, *Syt4*, *Reep2*, and *Sil1* can be tentatively proposed as potential candidate genes for the *ihs* locus.

## Discussion

Spontaneous diabetic rodent models are invaluable tools for both understanding the pathogenesis of diabetes and identifying a novel diabetic gene. Our research group recently developed a novel insulin hyposecretion mouse that is suitable for identifying a novel gene involved in insulin secretion through regulating the Ca^2+^ signaling pathway [9]. This study showed that a recessively acting locus responsible for the hyposecretion of insulin in the *ihs* mouse was mapped to a 14.9-Mb region on chromosome 18 between *D18Mit233* and *D18Mit235* (Fig 2B), a region that contained 199 genes. This region overlapped the *Nidd2*, a NON-derived diabetogenic locus that controls blood glucose [22, 23].

We also found that *D4Mit13* showed a significant association between genotype and glucose tolerance (Table 1) and an LRS peak beyond suggestive threshold level in QTL analysis (Tables 2 and S6). This region overlapped the *Nidds* locus, which is associated with hyperglycemia and decreased β-cell mass [24, 25]. Furthermore, we identified a significant QTL peak (LRS = 13.1) of the blood glucose levels at 120 min after OGTT at *D11Mit38* on chromosome 11 (S6 Table). This region overlapped the *Nidd1n* locus, which is associated with glucose tolerance and fasting and non-fasting blood glucose levels in NSY mice [26]. Given the consecutive distribution of blood glucose levels in N_2_ progeny (Figs 1C and S1) and a considerable difference in the blood glucose levels at 120 min after OGTT between *ihs* (Fig 1A) and B6-*ihs* (Fig 3A) mice, the *Nidds* locus on chromosome 4 and/or the *Nidd1n* locus on chromosome 11 might have an influence on the clearance of blood glucose after glucose loading in *ihs* mice.

With respect to WGS analysis on the *ihs* genome, two *ihs*-specific nsSNVs in the *ihs* locus were identified. PROVEAN predicted that both p.N228S in *Slc25a46* and p.T529I in *Tcerg1* would be harmful to protein function (Table 3). In particular, p.N228S in *Slc25a46* was detected in only the *ihs* mouse but not in the ICGN mouse from which the *ihs* mouse was derived (Table 3). Slc25a46 is a member of the mitochondrial solute carrier 25 protein family and is proposed to play a role in mitochondrial dynamics and maintenance of cristae by interacting with mitochondrial proteins involved in mitochondrial fusion and fission such as optic atrophy1 (Opa1), mitofusin 1 (Mfn1), and mitofusin 2 (Mfn2), and by interacting with the mitochondrial contact site (MICOS) complex involved in the maintenance of cristae [27]. Although the substrates of Slc25a46 have not been identified [28], dysfunction of this gene results in neuropathy including Charcot-Marie-Tooth type 2 [29], Leigh syndrome [30], and optic atrophy [31]. Mitochondria play an important role in insulin secretion [32]. Mitochondria produce ATP by glucose metabolism, resulting in closing the ATP-sensitive potassium channels causing β-cell plasma membrane depolarization followed by an influx of Ca^2+^ and insulin granule exocytosis. Therefore, *Slc25a46* might be involved in the regulation of insulin secretion [32].

Tcerg1 is a nuclear protein that regulates transcription and pre-mRNA splicing by interacting with RNA polymerase II and pre-mRNA splicing factor SF1 [33, 34]. It has been suggested that this gene is directly and/or indirectly involved in regulating the expression of 900 or more genes [35]. Although the involvement of this gene in diabetes and insulin secretion has not been reported, Tcerg1 might indirectly affect insulin secretion through the regulation of insulin secretion related gene expression.

In addition to WGS analysis, a candidate gene approach was performed to identify the gene mutation responsible for hyposecretion of insulin in the *ihs* mice. Given the gene functions of the candidate genes at this locus, *Syt4*, *Pkd2l2*, *Reep2*, and *Sil1* genes were the candidates for the *ihs* gene. Syt4, a non-Ca^2+^ binding paralogue of the major β-cell Ca^2+^ sensor Syt7, interacts with Syt7, and is involved in controlling the Ca^2+^ sensitivity of insulin vesicle secretion during β-cell maturation. *Syn4*-deficient (*Syt4-/-*) mice showed a modest defect in glucose clearance and impaired GSIS. A significant higher basal GSISS was observed in pre-weaned *Syt4-/*- islets, implying that Syt4 increases the threshold of Ca^2+^ needed to trigger insulin secretion [36]. In addition, islet-specific overexpression of *Syt4* induced impairment of glucose tolerance during OGTT and impaired insulin secretion from isolated islets elicited by glucose [36]. RT-qPCR analysis of isolated islets in *ihs* mice showed a relative abundance of *Syt4* transcripts that was 1.3-fold higher than those in B6 (Fig 4B), suggesting *Syt4* gene is a potential candidate for the *ihs* mutation. Sil1 is a nucleotide exchange factor for the binding immunoglobulin protein (BiP), which is an endoplasmic reticulum chaperone [37, 38]. In the β-cells, Sil1 is involved in insulin biosynthesis and insulin secretion processes [39]. Furthermore, *Sil1*-deficient mice showed a phenotype similar to that of *ihs* mice, such as having severe impairment of glucose tolerance and GSIS, and normal insulin sensitivity [9, 39]. Reep2 belongs to the receptor expression enhancing protein superfamily and enhances the functions of taste receptor type1 member 3 (T1R3), which is a sweet taste receptor, by interaction of these proteins [40]. In the β-cells, T1R3 has been reported to promote insulin secretion by facilitating the metabolic pathway of mitochondria and subsequently increasing ATP production, suggesting that Reep2 might affect insulin secretion via T1R3 [41]. A decrease of 30% and 42% in the expression levels of the *Sil1* and *Reep2* genes, respectively, were seen in *ihs* islets compared with B6 islets (Fig 4B), suggesting that *Sil1* and *Reep2* gene are also the potential candidate for *ihs* mutation. Pkd2l2 is a member of transient receptor potential (TRP) superfamily and functions as a Ca^2+^ permeable cation channel [42, 43]. Although the physiological roles of this gene are unclear, Pkd2l2 might participate in insulin secretion by regulating intracellular Ca^2+^ influx in β-cells. However, *Pkd2l2* transcripts could not be detected in either *ihs* or B6 islets (Fig 4A), and the *ihs-*specific variants changing amino acids could not be found on the *Pkd2l2* gene (S7 and S8 Tables), suggesting that the *Pkd2l2* gene is not responsible for the *ihs* locus.

Although the result of the WGS analysis showed no *ihs-*specific variants changing amino acids on exons of these genes (S7 and S8 Tables), the RT-qPCR analysis revealed that the *ihs* mice showed significantly higher expression levels of *Syt4* gene and significantly lower expression levels of *Sil1* and *Reep2* than B6 mice (Fig 4B), suggesting that the *ihs* mutation is located in the promoter or intron region of these candidate genes. In general, the frequency of variations in exons of the genes is much lower than that in promoters and introns. As the *ihs* mice are spontaneous mutants derived from ICGN mice, which provide an animal model for congenital nephrosis [9], there is no suitable control strain. Therefore, we focused on the variants on the exons of the genes in the *ihs* locus as a first step toward identifying the *ihs* mutation responsible for impaired insulin secretion. Although further study, such as comprehensive gene expression analysis in *ihs* islets using RNA sequencing, is required for a better understanding of the molecular pathology of the *ihs* mice, *Slc25a46*, *Tcerg1*, *Syt4*, *Reep2* and *Sil1* are tentatively proposed as the potential candidates for the *ihs* gene.

## Conclusions

The *ihs* locus was identified in the region on chromosome 18 between *D18Mit233* and *D18Mit235* (14.9-Mb). Furthermore, *Slc25a46*, *Tcerg1*, *Syt4*, *Reep2* and *Sil1* in this region are tentatively proposed as potential candidate genes for the *ihs* gene. This will be a focus of future studies in both mice and humans. The identification of the gene responsible for the impaired insulin secretion of the *ihs* mouse is expected to lead to further understanding of the regulation of the Ca^2+^ signaling pathway and insulin granule exocytotic machinery in pancreatic β-cells.

## Supporting information

S1 Fig Distribution of the blood glucose levels at 120 min after glucose loading in N_2_ progeny. (TIFF)

S2 Fig Raw data of agarose gel electrophoresis from Fig 4A. (TIFF)

S1 Table SSLP markers used for genetic mapping. (XLSX)

S2 Table SSLP and SNP markers used to create B6-*ihs* mice. (XLSX)

S3 Table The gene-specific primer sets using sequence analysis. (XLSX)

S4 Table RT-qPCR primer sets. (XLSX)

S5 Table SSLP markers with a LOD score of 3 or higher. (XLSX)

S6 Table SSLP markers with an LRS peak for QTL of the blood glucose levels at 120 min after OGTT. (XLSX)

S7 Table SNPs in the *ihs* locus. (XLSX)

S8 Table InDel mutations in the *ihs* locus. (XLSX)

## Abbreviations

T2D: type 2 diabetes
OGTT: oral glucose tolerance test
KCl: potassium chloride
QTL: quantitative trait loci
B6: C57BL/6NCr
AUC: area under the curve
SSLP: simple sequence length polymorphism
SNP: single nucleotide polymorphism
NGT: normal glucose tolerance
IGT: impaired glucose tolerance
cM: Centimorgan
LRS: likelihood ratio statistics
RT-qPCR: reverse transcription-quantitative polymerase chain reaction
Mb: Megabase
WGS: whole-genome sequencing
nsSNV: nonsynonymouse single-nucleotide variation
InDel: insertion deletion
GSIS: glucose stimulated insulin secretion
