# Supplementary figures and images for: Genetic locus responsible for diabetic phenotype in the insulin hyposecretion (ihs) mouse
Source: PLoS One. 2020 Jun 5;15(6):e0234132. doi: 10.1371/journal.pone.0234132 (PMC7274380; doi:10.1371/journal.pone.0234132)

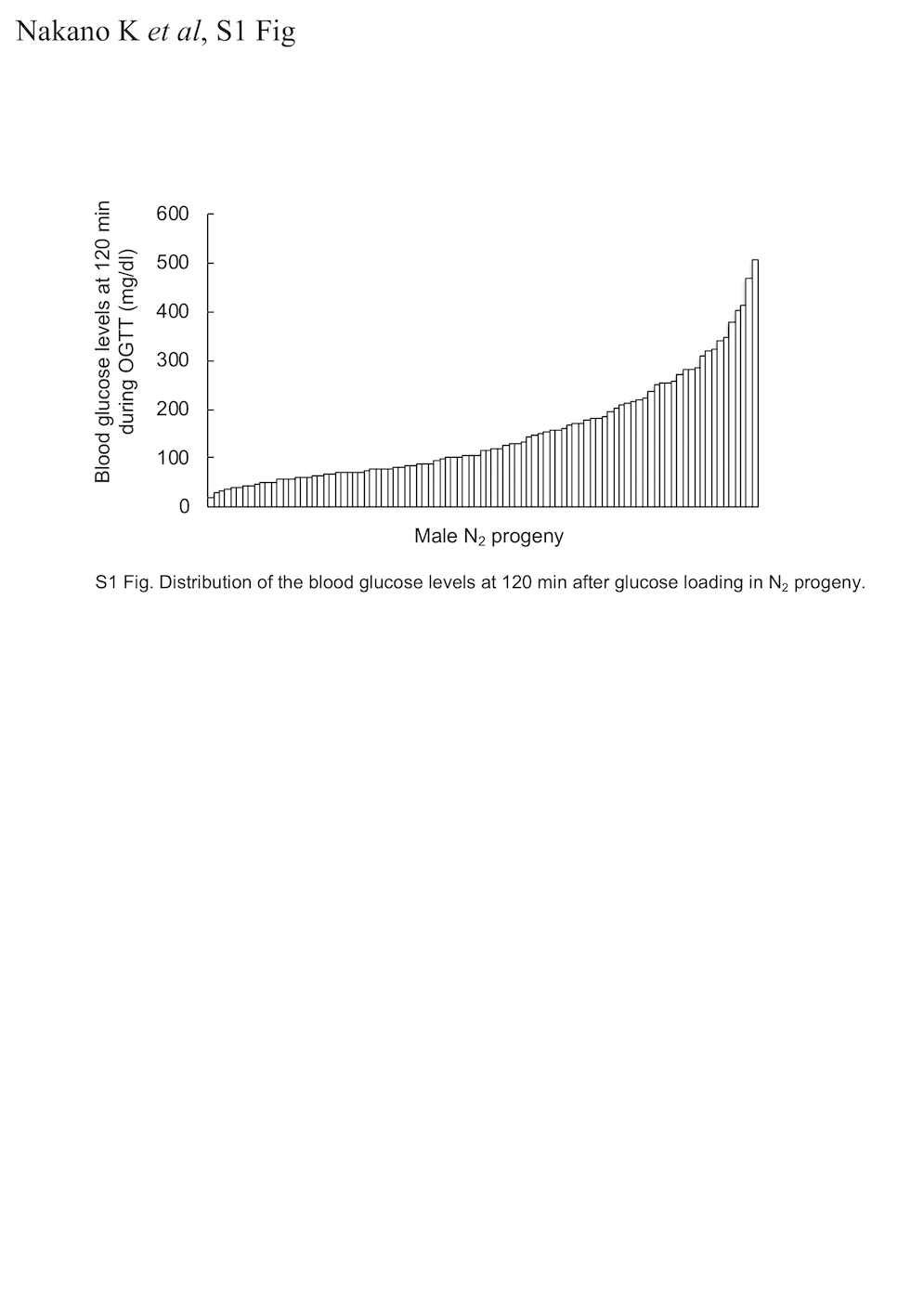

Supplement: S1 Fig — (TIFF) [file pone.0234132.s001.tiff]

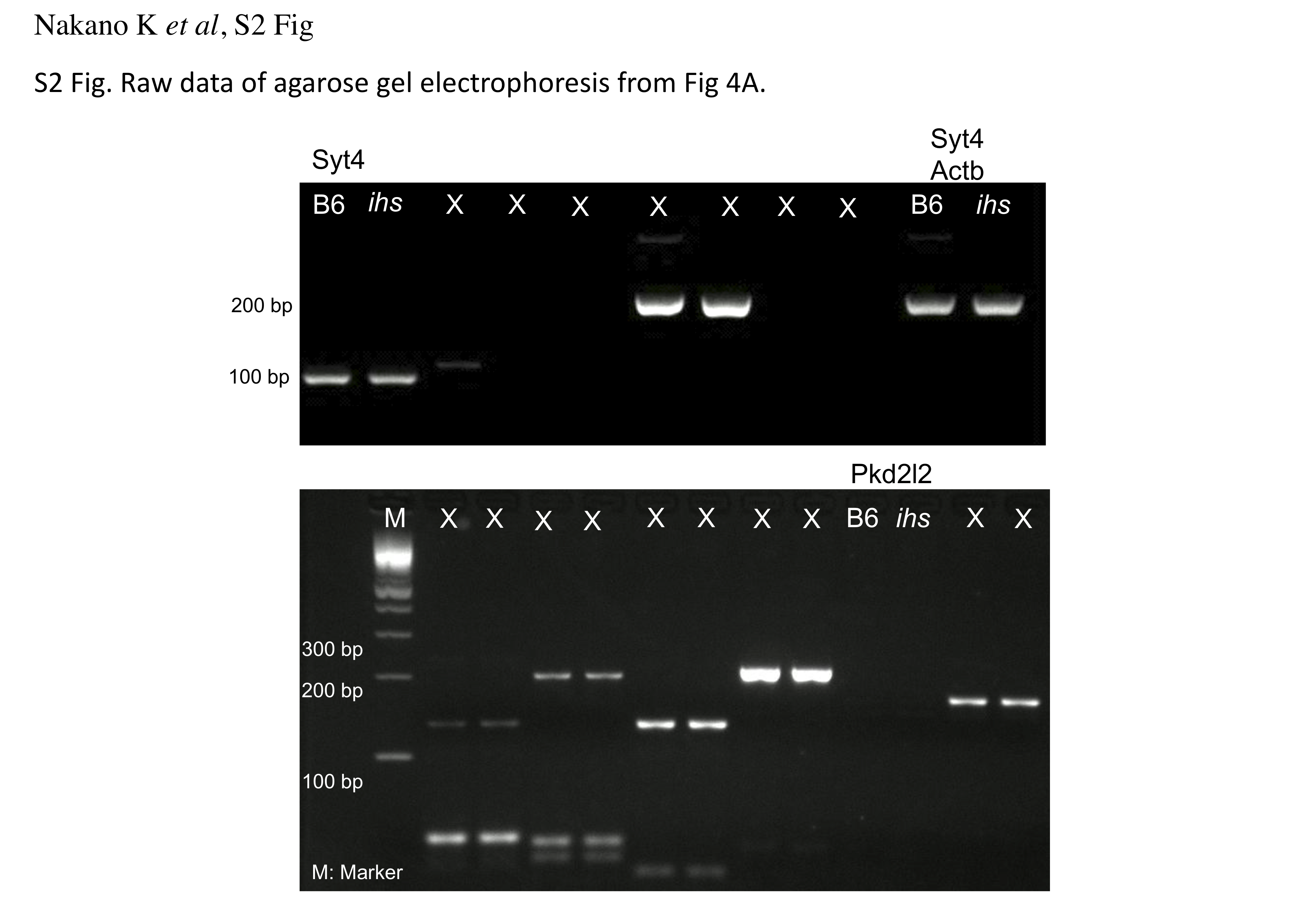

Supplement: S2 Fig — (TIFF) [file pone.0234132.s002.tiff]
